# Supplementary material for: Rosa davurica Inhibited Allergic Mediators by Regulating Calcium and Histamine Signaling Pathways
Source: Plants (Basel). 2023 Apr 6;12(7):1572. doi: 10.3390/plants12071572 (PMC10097250; doi:10.3390/plants12071572)
Supplement: Supplementary file 1 [file plants-12-01572-s001.zip › plants-2309572-supplementary.pdf]

Table S1. High-performance liquid chromatography (HPLC) profiling of *R. davurica* Pall. leaf extract (RLE) 210 nm, 254 nm, 280 nm, and 360 nm.

<table>
<tr><td>Instrument</td><td>Waters ARC system</td></tr>
<tr><td>Detector</td><td>Photodiode Array Detector (PDA)</td></tr>
<tr><td>Wavelength</td><td>210 nm, 254 nm, 280 nm, and 360 nm</td></tr>
<tr><td>Column</td><td>Atlantis T3 (250 mm*4.6 mm, 5 um)</td></tr>
<tr><td>Mobile phase</td><td>A: 0.1% Formic acid in Water
B: 0.1% Formic acid in Acetonitrile
<table>
<tr><th>Time (min)</th><th>A (%)</th><th>B (%)</th></tr>
<tr><td>0 - 5</td><td>97</td><td>3</td></tr>
<tr><td>5 - 15</td><td>97 – 90</td><td>3 - 10</td></tr>
<tr><td>15 - 20</td><td>90</td><td>10</td></tr>
<tr><td>20 - 25</td><td>85</td><td>15</td></tr>
<tr><td>25 - 30</td><td>85</td><td>15</td></tr>
<tr><td>30 - 40</td><td>80</td><td>20</td></tr>
<tr><td>40 - 45</td><td>80</td><td>20</td></tr>
<tr><td>45 -50</td><td>75</td><td>25</td></tr>
<tr><td>50 - 55</td><td>75</td><td>25</td></tr>
<tr><td>55 - 70</td><td>0</td><td>100</td></tr>
<tr><td>70 - 75</td><td>0</td><td>100</td></tr>
<tr><td>75 -80</td><td>97</td><td>3</td></tr>
</table></td></tr>
<tr><td>Flow rate</td><td>1.0 mL/min</td></tr>
<tr><td>Injection volume</td><td>10 μL</td></tr>
<tr><td>Temperature</td><td>40℃ (Oven), 15℃ (Autosampler)</td></tr>
</table>

Table S2. High-performance liquid chromatography (HPLC) of the ethyl gallate, ellagic acid, and *R. davurica* Pall. leaf extract (RLE)

<table>
<tr><td>Instrument</td><td>Waters ARC system</td></tr>
<tr><td>Detector</td><td>Photodiode Array Detector (PDA)</td></tr>
<tr><td>Wavelength</td><td>280 nm</td></tr>
<tr><td>Column</td><td>Atlantis T3 (250 mm*4.6 mm, 5 um)</td></tr>
<tr><td>Mobile phase</td><td>A: 0.1% Formic acid in Water
B: 0.1% Formic acid in Acetonitrile
<table>
<tr><th>Time (min)</th><th>A (%)</th><th>B (%)</th></tr>
<tr><td>0</td><td>85</td><td>15</td></tr>
<tr><td>10</td><td>85</td><td>15</td></tr>
<tr><td>15</td><td>82</td><td>18</td></tr>
<tr><td>20</td><td>82</td><td>18</td></tr>
<tr><td>25</td><td>0</td><td>100</td></tr>
<tr><td>30</td><td>0</td><td>100</td></tr>
<tr><td>30.1</td><td>85</td><td>15</td></tr>
<tr><td>35</td><td>85</td><td>15</td></tr>
</table>
</td></tr>
<tr><td>Flow rate</td><td>1.0 mL/min</td></tr>
<tr><td>Injection volume</td><td>10 μL</td></tr>
<tr><td>Temperature</td><td>40℃ (Oven), 15℃ (Autosampler)</td></tr>
</table>
